# Uncovering the research evolution and hotspots of metabolism in renal cell carcinoma over the last decade

**DOI:** 10.3389/fonc.2025.1537805

**Published:** 2025-08-26

**Authors:** Yifan Liu, Junzhe He, Donghao Lyu, Xinyue Yang, Haoyu Zhang, Siqi Tu, Yuntao Yao, Maodong Wei, Yuanan Li, Zihui Zhao, Runzhi Huang, Bingnan Lu, Xiao Xu, Xiuwu Pan

**Affiliations:** ^1^ Department of Urology, Xinhua Hospital Affiliated to Shanghai Jiao Tong University School of Medicine, Shanghai, China; ^2^ Department of Burn Surgery, The First Affiliated Hospital of Naval Medical University, Shanghai, China; ^3^ Department of Nursing, Xinhua Hospital Affiliated to Shanghai Jiao Tong University School of Medicine, Shanghai, China

**Keywords:** RCC, bibliometric analysis, metabolism, metabolic syndrome, microbiome, metabolic reprogramming

## Abstract

**Background:**

The vital role metabolism plays in RCC, a global disease with huge disease burden, has been widely acknowledged. However, bibliometric analysis remains underexplored in the context of metabolism in RCC.

**Methods:**

The Web of Science database was adopted to obtain relevant publications for further bibliometric analysis of countries, institutions, authors, journals, publications, references and keywords. Literature reading and keyword co-occurrence analysis were employed to figure out major points and hotspots in this field. The analysis was conducted by biblioshiny based on Bibliometrix package in R version 4.3.2.

**Results:**

From 15 May 2015 to 15 May 2025, 3010 relevant publications were retrieved. China was the most productive country and USA was the country with the highest total citations. The most productive institution was “Harvard University”. WANG Y published 46 publications with an H-index of 16. Core journals were identified with Bradford’s law. Additionally, three major points and hotspots were identified and discussed through bibliometric analysis and targeted literature reading.

**Conclusion:**

Our research provided a reference for future basic and clinical research through summarizing past research findings, analyzing current research hotspots, and prospecting the future development of research. “Metabolic alterations in RCC”, “Metabolic syndrome and RCC”, “Microbiome and RCC” were major points and hot spots in this field. In the future, a broader metabolic map could be made and more researches concerning metabolism during RCC treatment and drug resistance might bring more clinical significance.

## 1 Introduction

In 2022, kidney cancer ranked the 14^th^ globally, with about 434,419 new cases, causing 155,702 deaths (1). Renal cell carcinoma (RCC), accounting for over 90% of the malignancies in the kidney (2), refers to malignant neoplasm derived from the epithelium of renal tubule (3). The vital role that metabolism plays in RCC has been widely acknowledged (4, 5), and researches concerning metabolism in RCC has progressed quite fast in the past few years due to the advancement of understanding towards metabolism and related technologies.

People’s understanding towards “metabolism” has been continuously evolving and progressing. Around the 1960s, metabolism was generally viewed as a set of biochemical pathways. Krebs and colleagues identified the tricarboxylic acid cycle (6), laying the foundation for succeeding metabolic researches. During this period, most researches concerning metabolism in RCC focused on enzyme activity (7) and the levels of metabolites themselves (8). From the 1970s to the 1990s, breakthroughs in molecular biology and genetics, such as gas chromatography (9) and gene sequencing technology by Sanger (10) enabled scientists to connect metabolic pathways with gene regulation. This revealed how metabolism is precisely controlled at the genetic level. For example, researchers identified that abnormal CpG islands methylation in RCC might be involved in aberrant inactivation of VHL gene (11) and thus initiating metabolic alterations including upregulated glycolysis. Since 2000s, systems biology and high-throughput technologies, such as the next-generation sequencing (12), RNA sequencing (13) and single-cell RNA sequencing (14), have revolutionized metabolism research. Researchers began to view metabolism as an interconnected network intricately linked to cell fate determination, epigenetic regulation, and disease pathogenesis. The Warburg effect was reinterpreted in this context, revealing that cancer cells could gain survival advantages through metabolic reprogramming, such as the overexpression of the LDHA gene (15). Today, metabolism is widely recognized as a central regulator of cell fate, immunity, aging, and environmental sensing.

The advancement of metabolomics-related technologies and the evolving understanding of metabolism have evolved into the concept of metabolomics. In 1998, the term “metabolomics” was first put forward. It means the comprehensive study of all the small molecules in a biological sample, representing biochemistry on a large scale (16). The focus of metabolomic studies, as the advancement of technologies and people’s understanding, is transitioning from merely profiling chemicals or molecules to uncovering the biological narratives.

Metabolism in RCC is a fast-paced field, especially in recent years with the development of advanced concepts and technologies. Hence, it is of necessity to timely describe and summarize the knowledge structure and trend topics of this field in recent years. Bibliometric could provide us with a comprehensive landscape of a specific field in a statistical and quantitative manner, discovering hot spots and trend topics in a field. This effective analytical method has been applied in various filed, from breast cancer (17), prostate cancer (18) to lung cancer (19), etc. However, bibliometric analysis remains a void in the field of metabolism in RCC. Here, we tried to summarize past research findings, analyze current research hotspots, and prospect the future development of this field from 2015 to 2025. As a bibliometric study, we hope our work can serve as a foundational reference for future basic and clinical research in this field.

## 2 Materials and methods

### 2.1 Data sources and retrieval methods

This bibliometric study was mainly based on the Web of Science (WoS) database. Search term as follows was applied to collect data comprehensively from 15 May 2015 to 15 May 2025: ((TS = ((renal OR kidney) NEAR/2 (cancer* OR tumor* OR tumor* OR neoplasm* OR carcinoma* OR oncology))) AND ((TS = metabolism) OR (TS = metabolic) OR (TS = metabolome) OR (TS = metabolite) OR (TS = metabonomics) OR (TS = metabolomics))). A total of 3173 publications in Web of Science (Core Collection) were obtained and 3010 of them, comprising articles and reviews, were ultimately included in our study.

### 2.2 Bibliometric strategies

All data were integrated in one TXT file, and were uploaded to Biblioshiny, a web application based on Bibliometrix package (20) in R version 4.3.2 for further analysis. After systematically analyzing the data from the perspective of authors, countries, institutions, journals, publications, keywords, etc., a relatively comprehensive understanding of this field could be obtained. Here, we adopted number of publications and citations to provide a brief overview of this field. And H-index (21), which means H of one’s publications were cited at least H times, were used as a criterion for assessing both the quantity and quality of an author or a journal. The M-index of an author equals the H-index/the years since first publication (22). Publications of an author is ranked in decreased order of citations, and G-index is the largest sequence number (denoted as G) of a publication that has at least G^2^ citations (23). Bradford’s law (24) divides journals into several zones in a ratio of 1: k: k2: … (k > 1), with each of the zones accounting for equal publications in a certain field. The first zone with the least journals is perceived as core collections. Lotka’s law (25) states that the number of authors with one publication is n^2^ times as many as those with n publications. In co-citation network, the weight of lines between two articles indicates the frequency of which they are cited simultaneously. Similarly, keyword co-occurrence measures the correlation between keywords occurred in the identical publication. The topics could be classified into four groups by the thematic map with the “Density” axis representing the development degree and the “Centrality” axis signifying the relevance degree of these themes or fields: “Niche Themes” might be some small, specialized but well-developed territories with high “Density” but low “Centrality”. “Motor Themes” might be some core areas that received continuous attention in a field with both high “Density” and “Centrality”. “Basic Themes” might not be the hot spots at present, but served as corner stones in a field with high “Centrality” but low “Density”. “Emerging or Declining Themes” might refer to new ideas that existed for a relatively short period of time.

The parameters used during the analytical process were as follows:

The collaboration network between institutions adopted Walktrap algorithm with 30 nodes. The collaboration network between authors adopted Walktrap algorithm with 30 nodes. The co-citation network between publications adopted Walktrap algorithm with 50 nodes. The keywords co-occurrence network adopted Louvein algorithm with 50 nodes. The thematic evolution plot adopted Walktrap algorithm with 3 cutting points, namely 2019, 2021 and 2023. The remaining settings utilized custom settings, or could be intuitively seen from the plot.

## 3 Result

### 3.1 Annual publications

**Figure 1:**
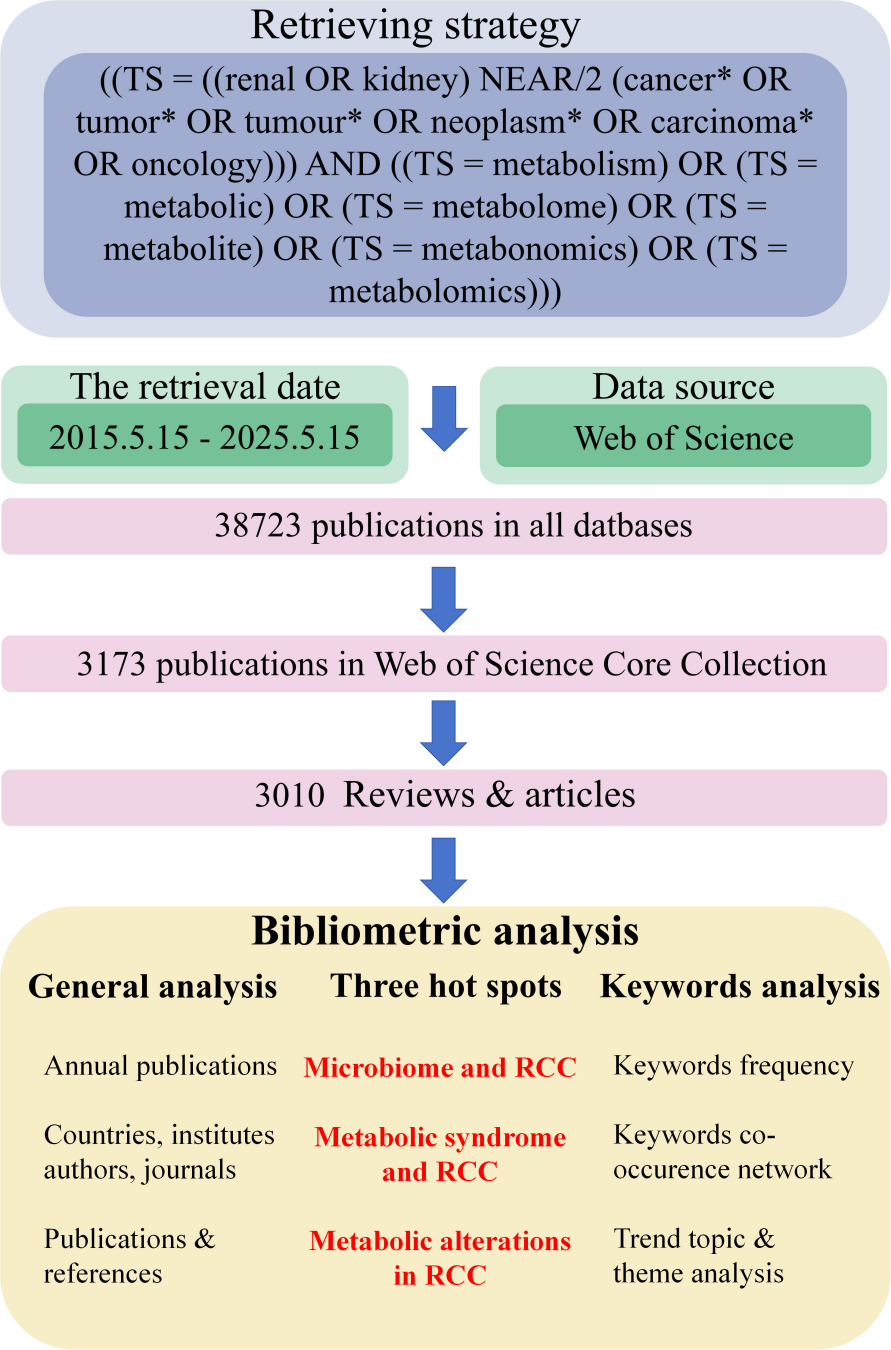
A simple flowchart of our study. Data source, researching process and bibliometrics analysis were demonstrated in this figure. RCC, renal cell carcinoma.

The complete retrieval and analytic process was shown in **Figure 1**. From 15 May 2015 to 15 May 2025, 3010 publications (reviews or articles) in Web of Science (core collection) were written by 16884 authors with an annual growth rate of 8.86%, published on 1002 journals. Annual scientific production could be utilized to partially measure the popularity of a specific field. As depicted in **Supplementary Figure S1**, the number of publications and citations manifested steady growth from 2015 to 2024, indicating that the field of “metabolism in RCC” is gaining more and more attention. Although the number of publications and citations seemed to drop in 2025, this might be attributed to the statistics ended at 15 May 2025, which only accounted for less than a half of the whole year.

### 3.2 Countries and institutions

**Figure 2:**
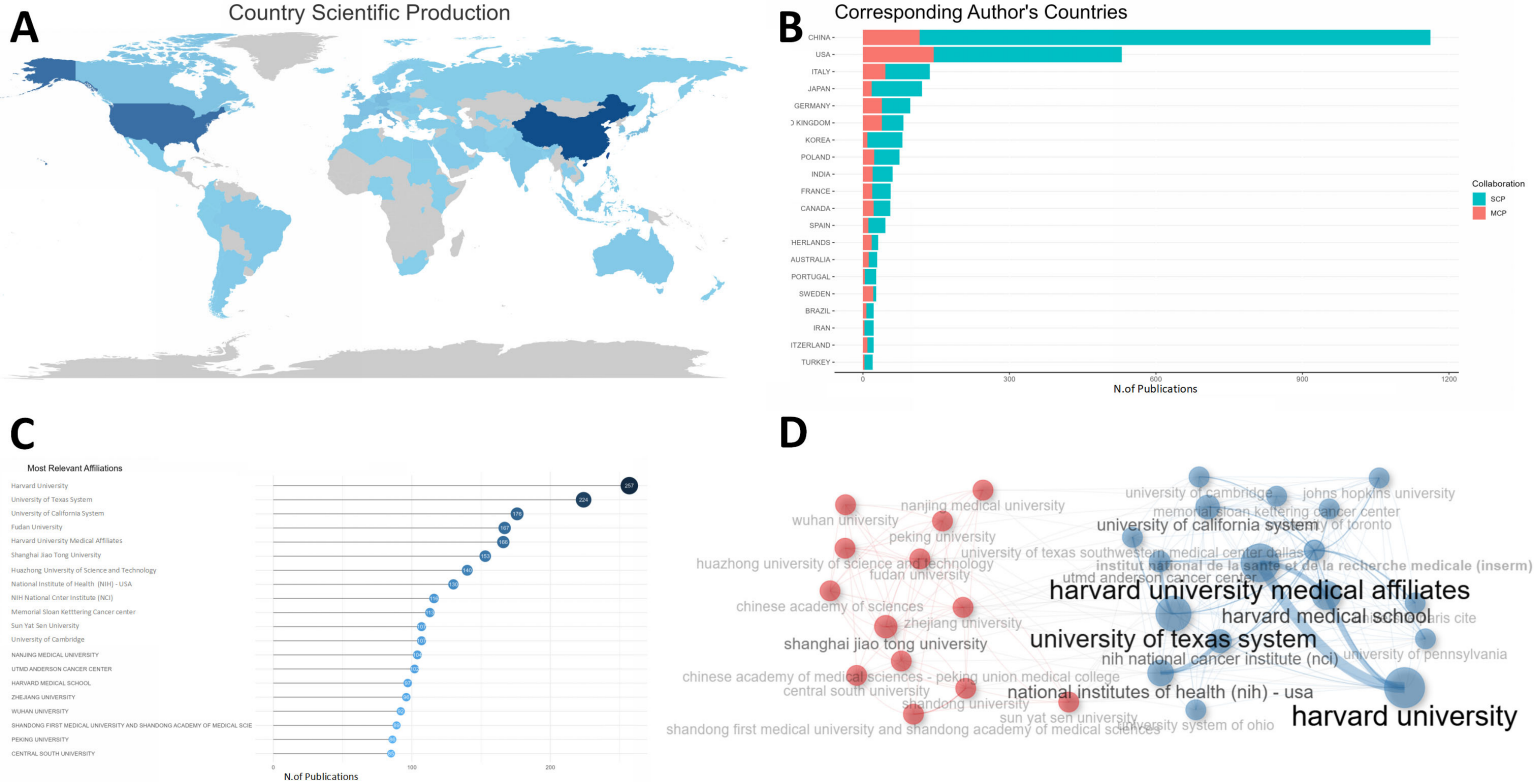
Country and institute analysis. **(A)** This world map shows the collaborations between different countries. The darkness of the country indicated the quantity of publications and the number of lines manifested cooperation between countries. **(B)** This plot shows the top 20 most productive countries measured by number of publications. SCP indicates researches conducted independently by scientists from the same country. MCP refers to studies completed collaboratively by scientists with different nationalities, indicating the degree of cooperation between countries. The USA, China, and ITALY were the top 3 countries with the highest MCP. **(C)** The top 20 most prolific institutions were listed. **(D)** The collaboration network manifested the relationships among different institutes in this field. SCP, single country publication; MCP, multiple countries publication; RCC, renal cell carcinoma.

A total of 66 countries contributed to this field. USA, China, ITALY were the top 3 among these countries according to total citations. The USA dominated the list with a total citation per item of 44.1, while China ranked second with a total citation per item of 17.0 (**Table 1**). Countries’ number of publications could be intuitively observed in **Figure 2A**, where the darkness of the country indicated the quantity of publications. China was the most productive country and USA, China and ITALY were also the top 3 countries with highest MCP (multiple countries publication), indicating that they were the countries with the most intention to cooperate (**Figure 2B**).

**Table 1:** The top 20 most contributing countries.

Country	TCs	MCP	Citation per item
USA	23355	145	44.1
CHINA	19758	116	17.0
ITALY	5892	46	43.0
UNITED KINGDOM	4097	39	49.4
GERMANY	2780	39	28.7
FRANCE	2027	19	35.6
CANADA	1854	22	33.1
JAPAN	1812	18	15.0
SPAIN	1590	11	34.6
SWEDEN	1547	21	57.3
POLAND	1495	23	19.9
MALAYSIA	1351	6	103.9
KOREA	1108	9	13.7
AUSTRALIA	1061	12	36.6
INDIA	1036	20	17.0
BRAZIL	797	7	36.2
NETHERLANDS	735	18	23.7
PORTUGAL	627	4	23.2
ISRAEL	605	3	55.0
IRAN	470	3	21.4

TCs, total citations; MCP, multiple countries publication.

As of institutions, **Figure 2C** showed the top 20 most prolific institutes, among which Harvard University in USA dominated the list with a production of 257 publications. Fudan University, Shanghai Jiao Tong University and Huazhong University of Science and Technology in China ranked fourth, sixth and seventh, respectively. In **Figure 2D**, the institutions could be classified into different groups, and each group had close relationships or common features, like publications they have contributed to, etc.

### 3.3 Authors

**Figure 3:**
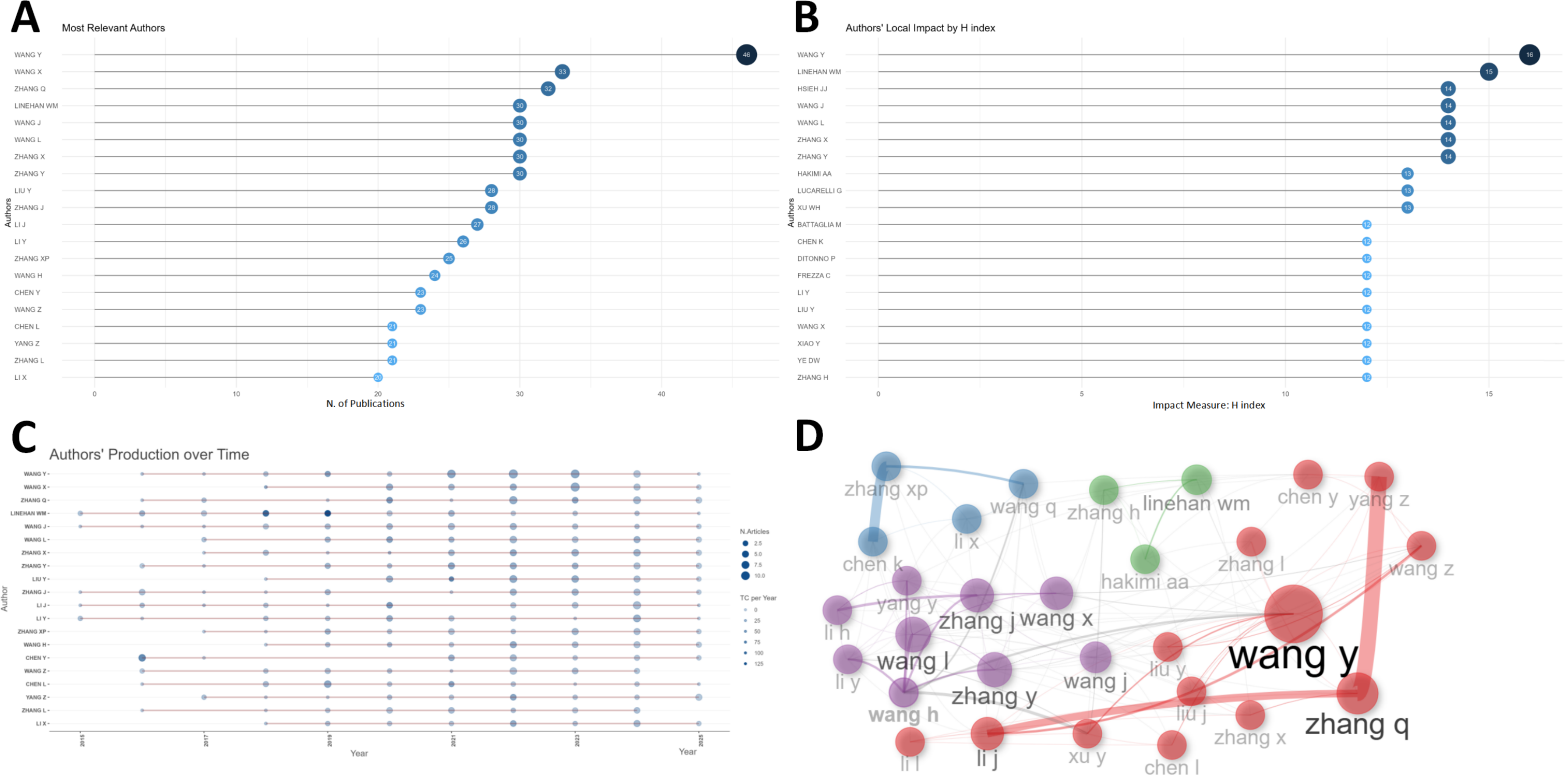
Author analysis. **(A)** The top 20 authors with most articles were listed in this plot. WANG Y and WANG X dominated the first and second place with 46 and 33 publications. **(B)** The top 20 authors with highest h-index were listed. WANG Y ranked first and LINEHAN WM ranked second. **(C)** The dynamic plot shows the timelines of the top 20 authors’ related researches of this field. The size and color-density of the nodes were positively correlated with the quantity of publication and total citation per year, respectively. **(D)** The collaboration network manifested the relationships among different authors in this field.

Up to 16884 authors contributed to this field. The top 20 most productive authors were listed in **Figure 3A**, where WANG Y and WANG X dominated the first and second place with 46 and 33 publications, surpassing other authors. As was shown in **Figure 3B**, the H-index of the top 20 authors varied from 12 to 16, with WANG Y still ranked first and LINEHAN WM ranked second (H-index = 15). In terms of local citations, HAKIMI AA topped the list with 648 local citations, closely followed by HSIEH JJ (**Supplementary Figure S2A**). LINEHAN WM dominated the list of G-index (**Supplementary Figure S2B**), but the list of M-index was dominated by “LAIMON YN” (**Supplementary Figure S2C**). **Figure 3C** presented the timelines of authors’ related researches in this field. “WANG Y” published 46 publications with an H-index of 16, indicating that “WANG Y” was the most productive and prominent author. The distribution of different authors with different number of publications was roughly in accordance with Lotka’s law (**Supplementary Figure S2D**). The authors could also be divided into different groups (**Figure 3D**). The top 20 most productive authors were also listed in **Table 2** with their number of publications, total citations, H-index, G-index and M-index.

**Table 2:** The top 20 most productive authors.

Rank	Authors	N. of publications	TCs	H-index	G-index	M-index
1	WANG J	46	901	16	29	1.6
2	WANG X	33	668	12	25	1.5
3	ZHANG Q	32	640	12	25	1.2
4	LINEHAN WM	30	2113	15	30	1.364
5	WANG J	30	464	14	21	1.273
6	WANG L	30	613	14	24	1.556
7	ZHANG X	30	536	14	23	1.556
8	ZHANG Y	30	567	14	23	1.4
9	LIU Y	28	692	12	26	1.5
10	ZHANG J	28	438	12	20	1.091
11	LI J	27	616	10	24	0.909
12	LI Y	26	473	12	21	1.091
13	ZHANG XP	25	363	11	18	1.222
14	WANG H	24	377	10	19	1.25
15	CHEN Y	23	799	9	23	0.9
16	WANG Z	23	259	10	15	1
17	CHEN L	21	552	11	21	1.1
18	YANG Z	21	316	9	17	1.000
19	ZHANG L	21	206	9	13	0.900
20	LI X	20	292	10	17	1.250

TCs, total citations.

### 3.4 Journals

**Supplementary Figure S3A** demonstrated the top 20 journals extracted from 1002 journals according to number of publications, Local citations, total citations and H-index were also adopted to evaluate journals’ impact, as is shown in **Supplementary Figures S3B–D**. **Supplementary Figure S3E** displayed 41 core journals according to the Bradford’s law in the field of metabolism in RCC.

Detailed information of the top 20 journals with highest number of publications, including their impact factor, Quartile in category, H-index and total citations, was shown in **Supplementary Table S1**.

### 3.5 Publications and references

**Figure 4:**
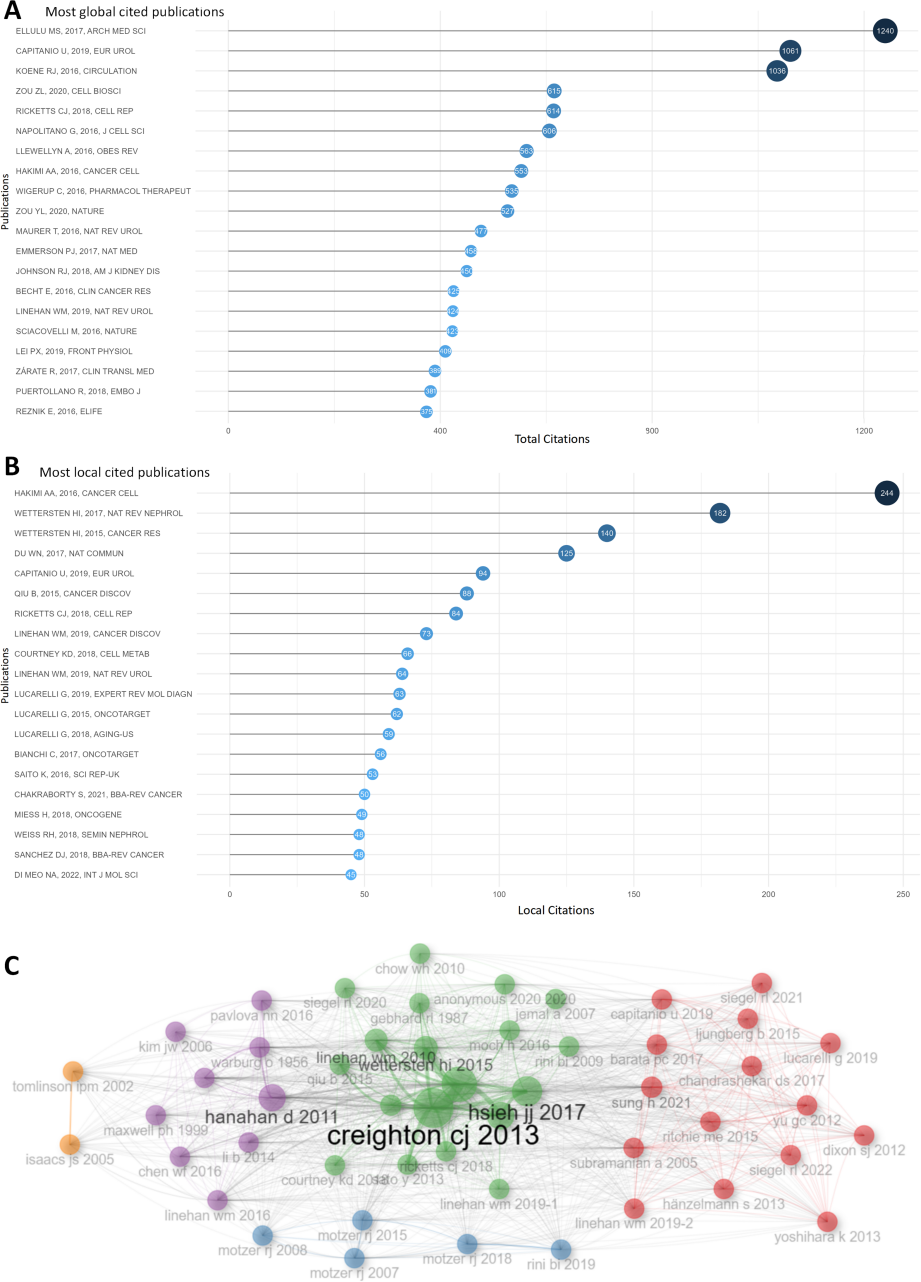
Publication analysis. **(A)** The top 20 publications were ranked according to the total citations. **(B)** The top 20 publications were listed according to local citations. **(C)** This plot demonstrates the publications clusters. Co-cited publications were put in the same cluster with identical color, and different clusters of publications might indicate different hot spots in this fields.

**Figure 4A** demonstrated the top 20 publications based on total citations, *Obesity and inflammation: the linking mechanism and the complications* (total citations = 1240), *Epidemiology of Renal Cell Carcinoma* (total citations = 1061), *Shared Risk Factors in Cardiovascular Disease and Cancer* (total citations = 1036) had the most total citations, surpassing other publications whose total citations were all below 1000. **Figure 4B** presented the top 20 publications with the largest local citations, *An Integrated Metabolic Atlas of Clear Cell Renal Cell Carcinoma* with local citations of 244 topped the list, while local citations of other publications were all lower than 200. Co-cited publications were put in the same cluster with identical color, and different clusters of publications might indicate different hotspots in this field (**Figure 4C**).

As for references, **Supplementary Table S2** illustrated the top 20 references out of more than 144,000 references in total according to local citations. *Comprehensive molecular characterization of clear cell renal cell carcinoma*, *An Integrated Metabolic Atlas of Clear Cell Renal Cell Carcinoma*, *Hallmarks of cancer: the next generation* were the top 3 in the list, indicating their fundamental impact in this fields. These publications could probably be regarded as the cornerstone of this fields.

### 3.6 Keywords, trend topic and themes analysis

Keywords could be regarded as the essence of publications, which concisely summarized major topics and main information of a publications. A total of more than 6000 keywords were extracted from our retrieval data. “Cancer”, “expression”, “metabolism”, “renal cell carcinoma” were the top 4 keywords with frequencies over 400, while those of other keywords were lower than 200, which could also be intuitively observed in **Supplementary Figure S4A**.

**Figure 5:**
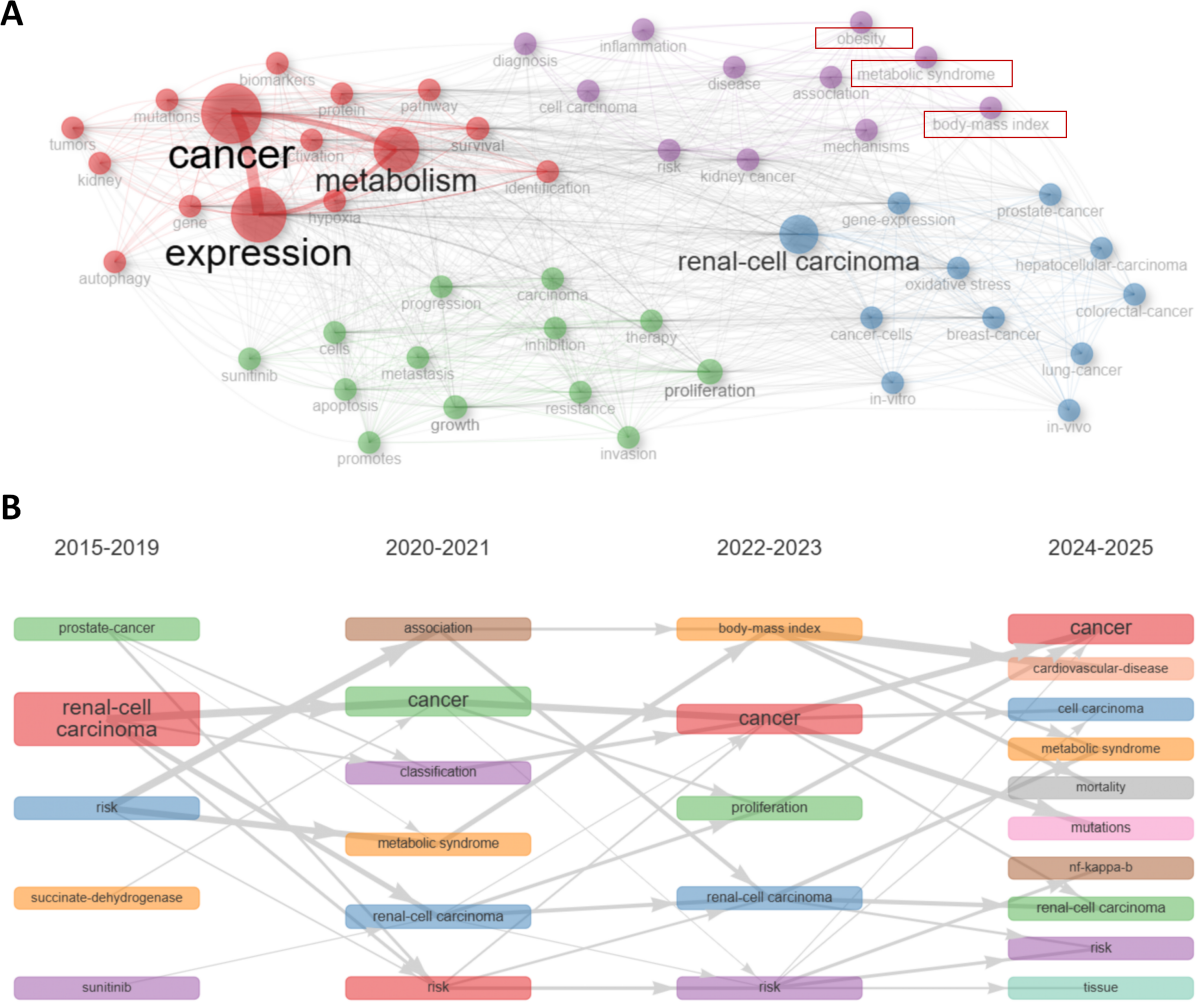
Keyword and hot spot analysis. **(A)** Keywords were distributed in different clusters in the keyword co-occurrence network. The red cluster indicated metabolic alterations in RCC. The purple cluster focused possibly on the correlations between metabolic syndrome and RCC. The green cluster mainly reflected therapy techniques and potential mechanisms. The blue cluster might focus on some shared mechanisms among RCC and other cancer types. **(B)** This dynamic plot demonstrated the thematic evolution. “metabolic syndrome”, as well as its related term “body-mass index”, were emerging keywords recent years from 2020, indicating metabolic syndrome and RCC might be a hotspot of this field recent years.

In keyword co-occurrence network, as is shown in **Figure 5A**, the red cluster was characterized by terms “metabolism”, “expression”, “cancer”, “activation”, “hypoxia”, “mutations”, indicating metabolic alterations in RCC and their influences on RCC cells. The purple cluster focused possibly on the correlations between metabolic syndrome and RCC, as terms like “metabolic syndrome”, “body-mass index”, “obesity”, “kidney cancer” could be seen in this cluster. The green cluster mainly reflected therapy techniques and potential mechanisms in this field, as terms like “sunitinib”, “therapy” could be observed. The blue cluster might focus on some shared mechanisms among RCC and other cancer types. We would make further explorations in the discussion part later about the red and purple clusters.

Trend shifts in a particular field could be succinctly presented by keywords dynamics. As can be observed in **Figure 5B**, “metabolic syndrome”, as well as its related term “body-mass index”, were emerging keywords recent years from 2020, indicating metabolic syndrome and RCC might be a hotspot of this field recent years. Development of trend topics overtime was shown in **Supplementary Figure S4B**. The classified topics were displayed in **Supplementary Figure S4C** by a thematic map.

## 4 Discussion

RCC is a global disease with heavy burden, and metabolism is considered to play vital roles in RCC. The concept of metabolism and related technologies have undergone rapid development, which has led to significant progress in the field of “metabolism in RCC”. However, bibliometric analysis in this field remains a void. Therefore, we conducted this study, hoping to provide a foundational reference for future basic and clinical research in this field.

In this study, we tried to summarize past research findings, analyze current research hotspots, and prospect the future development of this field by reading and analyzing crucial articles and reviews from 2015 to 2025 in the Web of Science (Core Collection) with the help of bibliometric methods.

### 4.1 General information

The annual publications and citation in this field increased steadily from 2015 to 2024 with a drop in 2025, possibly for the statistic end point of 15 May 2025. As for countries, USA, China and UK were in the top 3 list according to total citations, and they were also the countries with most cooperation as indicated by their high MCP. For institutions’ productivity, Harvard University in USA dominated the list. Fudan University, Shanghai Jiao Tong University and Huazhong University of Science and Technology in China ranked fourth, sixth and seventh, respectively. As for authors, WANG Y dominated the list of productivity and H-index, while HAKIMI AA, LINEHAN WM, LAIMON YN ranked the first in terms of local citations, G-index, M-index, respectively. Journals were analyzed based on number of publications, total citations, local citations and h-index. Core journals were identified with Bradford’s law. Publications on these journals would be conductive to keep pace with the latest development in this field, as well as obtain basic knowledge in this field. After analyzing the keyword co-occurrence network, combined with targeted literature reading, we identified several major points and hotspots over the past decade in the field of metabolism in RCC, namely the metabolic alterations in RCC, the metabolic syndrome and RCC, the microbiome and RCC.

### 4.2 Metabolic alterations in RCC

#### 4.2.1 Metabolic gene reprogramming in RCC

A series of genes were involved in metabolic reprogramming in RCC. Generally, RCC could be majorly classified into three subtypes: clear cell RCC (ccRCC), which was the most prevalent (26), papillary RCC (pRCC), and chromophobe RCC (chRCC). Apart from these, there were some rare subtypes, such as medullary RCC, collecting duct RCC, and hereditary leiomyomatosis renal cell cancer (HLRCC) (2, 26–28).

VHL gene mutation and subsequent HIF accumulation (4, 11) are widely recognized as the most common pathogenic events in ccRCC. HIF act as a transcriptional regulator that promote the expression of a range of glycolysis-related genes, including GLUT1 (glucose transporter-1), PGK (phosphoglycerate kinase), LDHA (lactate dehydrogenase), PDK1 (pyruvate dehydrogenase kinase), and HK (hexokinase) (29, 30). Besides, HIFs could also contribute to the downregulation of TCA cycle and oxidative phosphorylation (31). Genes involved in PI3K/AKT/mTOR signaling pathway, including PTEN, TSC1/2, and PIK3CA (5, 32), were also commonly mutated in ccRCC based on TCGA database (33). Notably, mTORC1 could augment the expression of HIF (34). In ccRCC cells, MYC was another commonly overexpressed gene, which might function during metabolic reprogramming of glutamine and fatty acid synthesis (35–37).

As for other cancer types, FH was regarded as the gene responsible for HLRCC (38), SDH was found to be associated with familial renal cancer (4), and MET was thought to be correlate with hereditary pRCC (4).

#### 4.2.2 Carbohydrate metabolism

Glycolysis was upregulated in ccRCC cells. GLUT-1 was upregulated (29), which subsequently led to increased glucose uptake. After entering the cancer cell, glucose was further catalyzed into G-6-P by upregulated HK (15). Other enzymes involved in glycolysis or subsequent lactate fermentation, including GPI, PGK, LDH-A, etc. were also upregulated (15), further substantiated the upregulation of glycolysis. By upregulated enzyme G6PD (39), G-6-P could then enter Pentose Phosphate Pathway (PPP), where NADPH and ribose sugars were produced. TCA and ETC was downregulated in RCC cells. Two critical enzymes, PDH and PC, which help pyruvate to enter TCA cycle, were downregulated in RCC (40, 41), implicating a decreased shunt flux into TCA cycle (40). However, citrate, an intermediate in TCA, was upregulated (42, 43). Downregulation of oxidative phosphorylation complexes (II, III, IV) of the respiratory chain and ATP synthase was associated with RCC (44), implicating downregulation of ETC.

Metabolic alterations in carbohydrate indicate that RCC tends to use glycolysis rather than oxidative phosphorylation to survive, which was in consistent with the definition of Warburg effect (45). The Warburg effect enabled cancer cells to thrive in a nutrient-deficient condition (46), while the PPP was utilized to antagonize oxidative stress (47) as well as providing nucleotides (46).

#### 4.2.3 Lipid metabolism

Lipogenesis was upregulated in ccRCC. Actually, lipids storage was not only a morphological manifestation of ccRCC, but also an indicator of malignancy. Recently, mesoderm induction early response 2 (MIER2) was identified as a new biomarker for RCC, which could promote malignancy and sunitinib resistance by inducing lipids accumulation in RCC (48). This further indicated the vital role of lipid metabolism.

##### 4.2.3.1 Fatty acid metabolism

Various alterations contributed to fatty acid synthesis in ccRCC. Stearoyl-CoA desaturase 1 (SCD1), an enzyme that elongates and desaturates fatty acids to produce unsaturated fatty acids like triglycerides and phospholipids (49), was upregulated in ccRCC tissues (50). Compared with non-malignant renal tissue, the mRNA level of ACC, the rate-limiting enzyme during FA synthesis (51), was upregulated in ccRCC and had a positive correlation with unfavorable overall survival (52). Besides, the IHC staining intensity of FASN, the terminal enzyme in *de novo* FA synthesis, was shown to be positively correlated with tumor staging and distant metastasis in 120 RCC patients (53). Besides, the expression and activity of Carnitine palmitoyl transferase 1A (CPT1A), which functions to transport lipids into mitochondria for further oxidative degradation (54), was down regulated in ccRCC (55). Moreover, after lipids were produced and protected from degradation, another protein called Perilipin 2 (PLIN2) would promote lipids storage. PLIN2, a protein on the surface of lipids droplets (56), was proved to be over-expressed in ccRCC patients’ samples and was shown to promote lipids storage, tumor progression and tumor proliferation in ccRCC xenografts (57).

Apart from alterations in lipids metabolism, many altered metabolites in carbohydrates and amino acids metabolism could influence lipids synthesis. Citrate, which was upregulated in TCA cycle, could generate intracellular acetyl-CoA via the action of ATP citrate lyase (ACL) (58) for further fatty acid synthesis. Interestingly, ACL was observed to be upregulated in RCC cells (59), and knockdown of ACL could inhibit proliferation and induce apoptosis in RCC cells. In fact, citrate could also be obtained from reductive carboxylation of glutamine (60, 61), where amino acid metabolism showed its importance during lipids synthesis.

##### 4.2.3.2 Upregulated cholesteryl ester metabolism

CE metabolism was upregulated in ccRCC (62–64), and inhibition of 3 beta-hydroxy steroid dehydrogenase type 7 (HSD3B7), an enzyme concerning CE metabolism, might induce apoptosis in ccRCC cells (65). Mechanically, CE could be oxidized to 7α-hydroxycholesterol (7α-OHC), an intermediate product triggering apoptosis, and HSD3B7 could catalyze 7α-OHC into cholic acid or chenodeoxycholic acid (CDCA), and thus prevent apoptosis (65).

##### 4.2.3.3 Clinical significance of lipids metabolism in ccRCC

Metabolic reprogramming of lipids made it a promising endeavor to adopt lipids as biomarkers for ccRCC diagnosis or prognosis. In fact, researchers have found by lipidomics and machine learning that a 26-lipids panel could have excellent performance in distinguishing stage I and II with stage III and IV ccRCC (66). In another study focusing on using metabolites to predict ICI response, researchers found that most of the selected marker metabolites (9 in 10) were very-long-chain FAs (67). Furthermore, in 2025, another group of researchers developed a fatty acid metabolism signature (68) based on TCGA and GEO database, which could effectively predict the survival and response to targeted therapy and immunotherapy among ccRCC patients. This further validated the vital role lipids metabolism played in ccRCC.

#### 4.2.4 Amino acid metabolism

Upregulated glutamine metabolism (42) might prevent RCC from cell death. GSH generated in this pathway could act to hinder ROS, hence protecting the cancer cells from oxidative stress (40). Interestingly, inhibition of GSH synthesis could induce ferroptosis in ccRCC cells (VHL- deficient) (69), and ferroptosis is characterized by lipid-peroxidation (70).

Increased downstream metabolites in Kynurenine pathways, including kynurenine and quinolinate, was identified with decreased tryptophan level in RCC (40), and IDO, which functions during tryptophan catabolism, was upregulated in endothelial cells of RCC compared with normal tissues (71). These implied an upregulated Kynurenine pathway, whose downstream metabolites might have immunosuppressive effects (40). Furthermore, the combined administration of IDO inhibitor and IFN-α was shown to inhibit RENCA cell (renal cancer cell line in mice) while IFN-α or IDO inhibitor along couldn’t inhibit tumor growth (71). Besides, *in vitro* experiment also indicated that DCs treated by IDO inhibitor, namely the epacadostat, could possibly enhance the oncolysis function of T cells (72).

ASS1, the key enzyme during arginine synthesis, was downregulated in ccRCC patients’ biopsy samples (73), indicating an arginine dependency in cancer cells. Arginine deprivation was thought to be a promising therapy for ccRCC (5).

### 4.3 Metabolic syndrome and RCC

Metabolic syndrome was defined by WHO as a pathological condition consisting abdominal obesity, insulin resistance, hypertension, and hyperlipidemia (74). A growing body of evidence suggested a close association between metabolic syndrome and RCC. Metabolic syndrome has already been identified as an independent prognostic factor in localized ccRCC patients (75) back to 2019, and a meta-analysis (76) composed of six studies in 2025 reconfirmed its independent correlation with RCC. In addition, some specific diseases related to metabolic syndrome, including diabetes, obesity and hypertension was discovered to be correlated with RCC as well. Diabetes was identified as an independent risk factor for TNM staging in ccRCC patients (77). Obesity was a well-recognized risk factor for RCC, and BMI was positively correlated with the incidence (78, 79) and pathological upstaging (80) of RCC. Hypertension was identified as an independent prognostic factor for RCC survival (81), and a positive link between both diastolic blood pressure and systolic blood pressure and RCC risk (82) was discovered.

Mechanically, hyperinsulinemia was thought to be the shared potential mechanism between diabetes, obesity and RCC. Notably, hyperinsulinemia was not only seen in diabetes, but also existed in in obesity state (83). Insulin can enhance IGF-1 synthesis and activation. Both insulin and IGF-1 have the effects of fostering cell proliferation and restraining apoptosis (84). Upon activation of insulin receptors (INSR) and IGF1 receptors (IGF1R), a series of signaling pathways is triggered. These include pathways such as PI3K/AKT, cyclin D1, HIF-1α and VEGF. The activation of these pathways could contribute to key characteristics of cancer, including enhancing cell proliferation, stimulating angiogenesis, and diminishing apoptosis (85–87). However, some researchers observed a positive correlation between better prognosis and increased IGF-1 levels (88), which might suggest a more complicated mechanisms behind IGF-1 and RCC.

High level of glucose might play a role between diabetes and RCC by stimulating the metabolism of RCC cells (84). It has been proposed that hyperglycemia can enhance cancer cell proliferation via elevating the levels of protein kinase C (PKC) and peroxisome proliferator-activated receptors (PPARs), which can in turn accelerate cellular metabolism and thereby foster cell proliferation (89).

Pro-inflammatory factors and leptin might play roles between obesity and RCC. It was believed that chronic inflammation existed in adipose tissue under obesity state (90). Obesity-related chronic inflammation might promote tumor by releasing pro-inflammatory factors, such as IL-6 with anti-apoptotic and cell proliferation effect via JAK2 and PI3K/AKT (91, 92), as well as TNF-α with anti-apoptotic effect via NF-kB (87). Notably, glutamine, which was correlated with decreasing inflammation and proinflammatory cytokines, was downregulated in obesity (93), which further implied the role of inflammation in mediating the link between obesity and RCC. Moreover, leptin, an adipocyte-specific protein functions to regulate satiety and bodyweight (94), might also promote RCC by promoting cell proliferation, upregulating VEGF and inhibiting apoptosis, possibly through HIF-1α and NF-κB (95). Leptin was also shown to be correlated with worse OS and migration of ccRCC cells (96).

Though the precise and detailed mechanism between RCC and hypertension has not been fully understood, some researchers (97) hypothesized that renin-angiotensin-system might play a potential role. Ang- (1-7) (generated from Ang I and Ang II) could promote xenograft tumor growth in nude mice and migration of caki-1 and caki-2 cell lines *in vitro* (98), and combined administration of sunitinib and telmisartan (a renin-angiotensin-system antagonist) was observed to induce more necrosis and less neo-angiogenesis in 786-O cell line xenograft in mice, indicating the potential role and therapeutic value of renin-angiotensin-system in RCC (99).

### 4.4 Microbiome and RCC

Studies have demonstrated that there were significant differences in the relative abundance of 20 gut bacteria species between RCC patients and control group (100). Besides, antibiotic use may reduce the efficacy of immunotherapy in metastatic renal cell carcinoma (101), yet using antibiotics to target Bacteroides spp. in stool can improve progression-free survival (PFS) in metastatic RCC patients receiving first-line VEGF-TKI therapy (102). Additionally, CBM588, a live bacterial product, enhance clinical outcomes in patients with metastatic renal cell carcinoma treated with nivolumab-ipilimumab (103). These findings suggest that microbiome may play significant roles in RCC.

The precise and comprehensive mechanism behind microbiome and RCC were not fully understood yet. However, there seemed to be some indirect evidences revealing potential mechanisms.

The microbiome may impact RCC through tryptophan metabolism. Some endogenous tryptophan metabolites can act as ligands for aryl hydrocarbon receptor (AhR) signaling (104), and AhR has been linked to the invasion of RCC (105). Researchers investigated the relationship between gut bacterial abundance and the expression levels of certain tryptophan metabolites (106). Kynurenic acid showed a negative correlation with the gut bacteria Prevotella-9 and Akkermansia, and kynurenine (Kyn) was found to enhance the migration and invasion of 786-O cells via AhR. These findings suggest that gut microbiota may activate AhR through its tryptophan metabolite kynurenine, thereby mediating RCC metastasis. The microbiome might also influence RCC by modulating immunity through lipid metabolism. In 2025, a research team (107) discovered that low intra-tumoral mycobiome abundance was associated with suppressed lipid catabolism, CD8+ T cell depletion, and poor prognosis. This implies that the intra-tumoral mycobiome might affect RCC via immune regulation mediated by lipid metabolism. Carbohydrate metabolism and related molecules may represent another target through which the microbiome affect RCC. SUCNR1, the receptor of succinate, an important molecule during TCA cycle, was linked to a variety of microbes, including beneficial bacteria possibly contributing to a better disease-specific survival rate in ccRCC (108). Additionally, taurine might also play a intermediate role between microbiome and RCC. Upregulated bacteria desulfovibrionaceae downregulated Lactobacillus was observed in ccRCC (100) while the serum level of taurine, which was considered to be consumed by bacteria desulfovibrionaceae (109) and promoted by Lactobacillus (110), also decreased in the ccRCC (100).

In the future, with the aid of continuously advancing new technologies, it may be possible for researchers to further map the spatial and temporal landscapes of metabolism in RCC, thereby more comprehensively elucidating the role of metabolism throughout the entire process of cancer initiation. Additionally, leveraging sophisticated omics technologies and the progress in microbiome-related research, it may be feasible in the future to construct a holistic metabolic map of the human-microbiome ecosystem, providing a more comprehensive understanding of the role of metabolism in renal cancer. Lastly, it is anticipated that more studies will focus on the role of metabolism in renal cancer treatment and drug resistance, revealing the close connection between metabolism and renal cancer from a more clinically significant perspective.

## 5 Conclusion

Through bibliometric analysis over 3010 articles and reviews, together with critical literature reading, our research summarized past research findings, analyzed current research hotspots, and prospected the future development in this field. Specifically, three major points were proposed. Firstly, the metabolic alterations in RCC. Metabolic reprogramming and the biological significance of metabolic changes were discussed from the perspective of carbohydrate, lipids and amino acid metabolism. Secondly, the metabolic syndrome and RCC. The correlation and potential mechanism between RCC and metabolic diseases, including obesity, diabetes, and hypertension were explored. Thirdly, the microbiome and RCC. Microorganisms may influence the development of RCC through specific metabolites, revealing a close connection between RCC and metabolism from a novel viewpoint. In the future, a broader metabolic map with spatial, temporal and human-microbiome interaction perspective could be made and more researches concerning metabolism’s role during RCC treatment and drug resistance might bring more clinical significance.

## 6 Limitations

In our study, publications from 2015 to 2025 in the field of metabolism in RCC were summarized by bibliometric analysis. Major points and hotspots over the past decade were explored and discussed. However, some flaws might still exist because of data acquisition method. All the publications were from Web of Science (core collection), and hence data from other sources were not included in our study. Besides, data after our retrieving date could not be included in our study as well. Data from multiple sources could be collected in future studies to make our understandings about this field more comprehensive and accurate. Moreover, this study aimed at providing a fundamental reference for future by summarizing major points and hotspots in this filed. Therefore, this study may not be able to provide conclusions with high level of evidence-based hierarchy like a meta-analysis.

## Data Availability

The original contributions presented in the study are included in the article/**Supplementary Material**. Further inquiries can be directed to the corresponding authors.
