# Supplementary material for: Uncovering the research evolution and hotspots of metabolism in renal cell carcinoma over the last decade
Source: Front Oncol. 2025 Aug 26;15:1537805. doi: 10.3389/fonc.2025.1537805 (PMC12417187; doi:10.3389/fonc.2025.1537805)
Supplement: Supplementary file 1 [file DataSheet1.pdf]

Supplementary materials

A

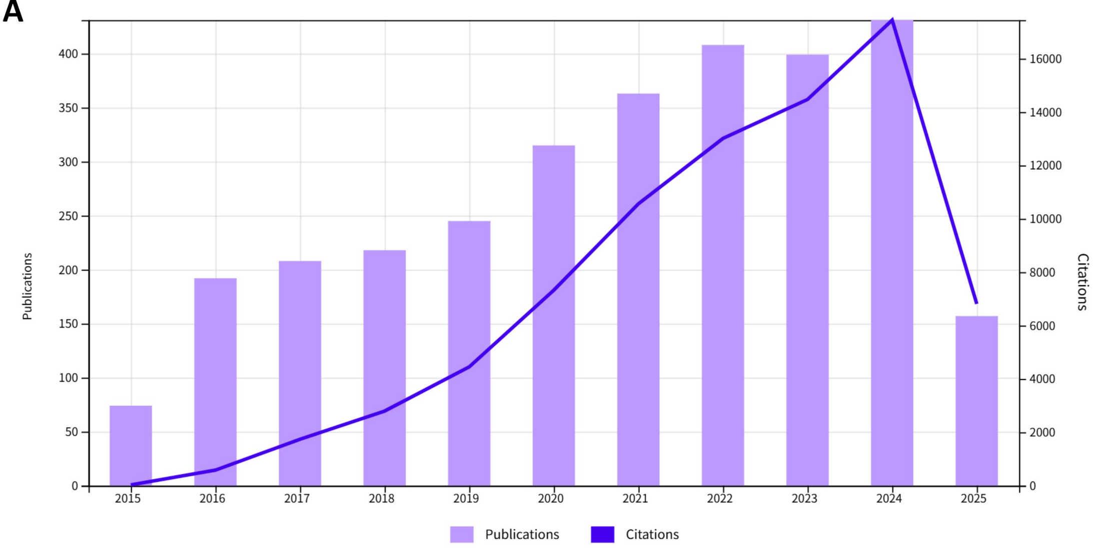

Figure S1. Annual publications and annual citations in the field "metabolism in RCC" from 2015 to 2025 were demonstrated in this plot.

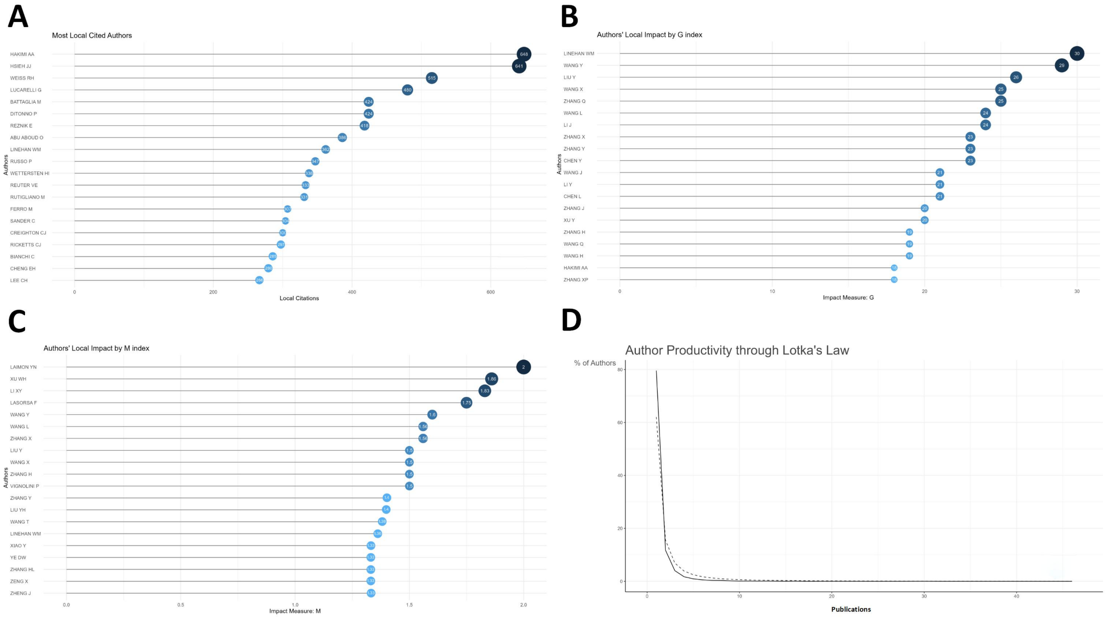

Figure S2. Author analysis. (A) The top 20 authors with highest local citations. (B) The top 20 authors ranked by G-index. (C) The top 20 authors with highest M-index. (D) The distribution of different authors with different number of publications was roughly in accordance with lotka's law.

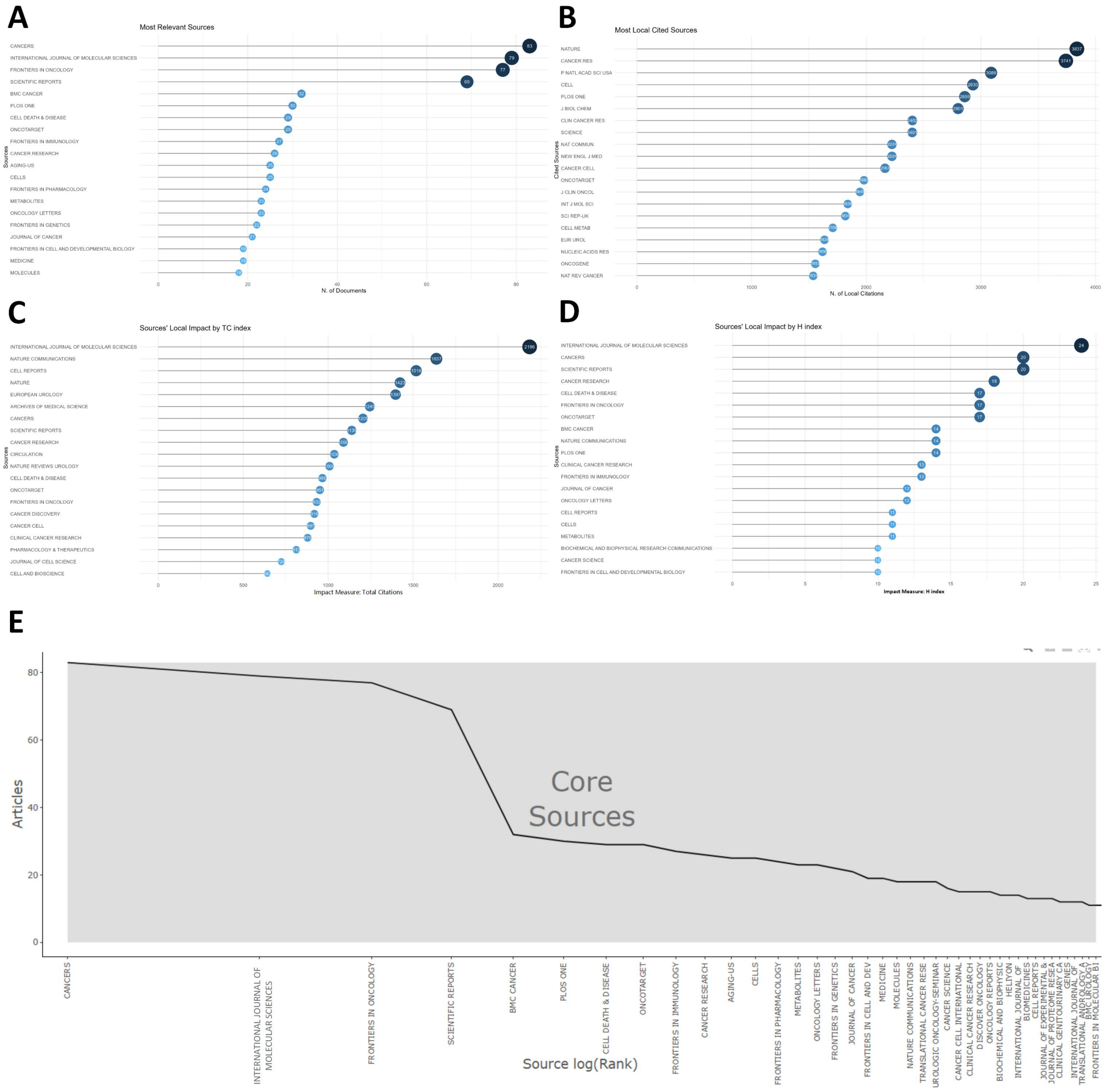

Figure S3. Journal analysis. (A) The top 20 journals were ranked according to number of publications. (B) The top 20 journals were listed according to local citations. (C) The top 20 journals were listed based on total citations. (D) The top 20 journals were ranked based on H-index. (E) The top 41 core journals were extracted according to the Bradford’s law.

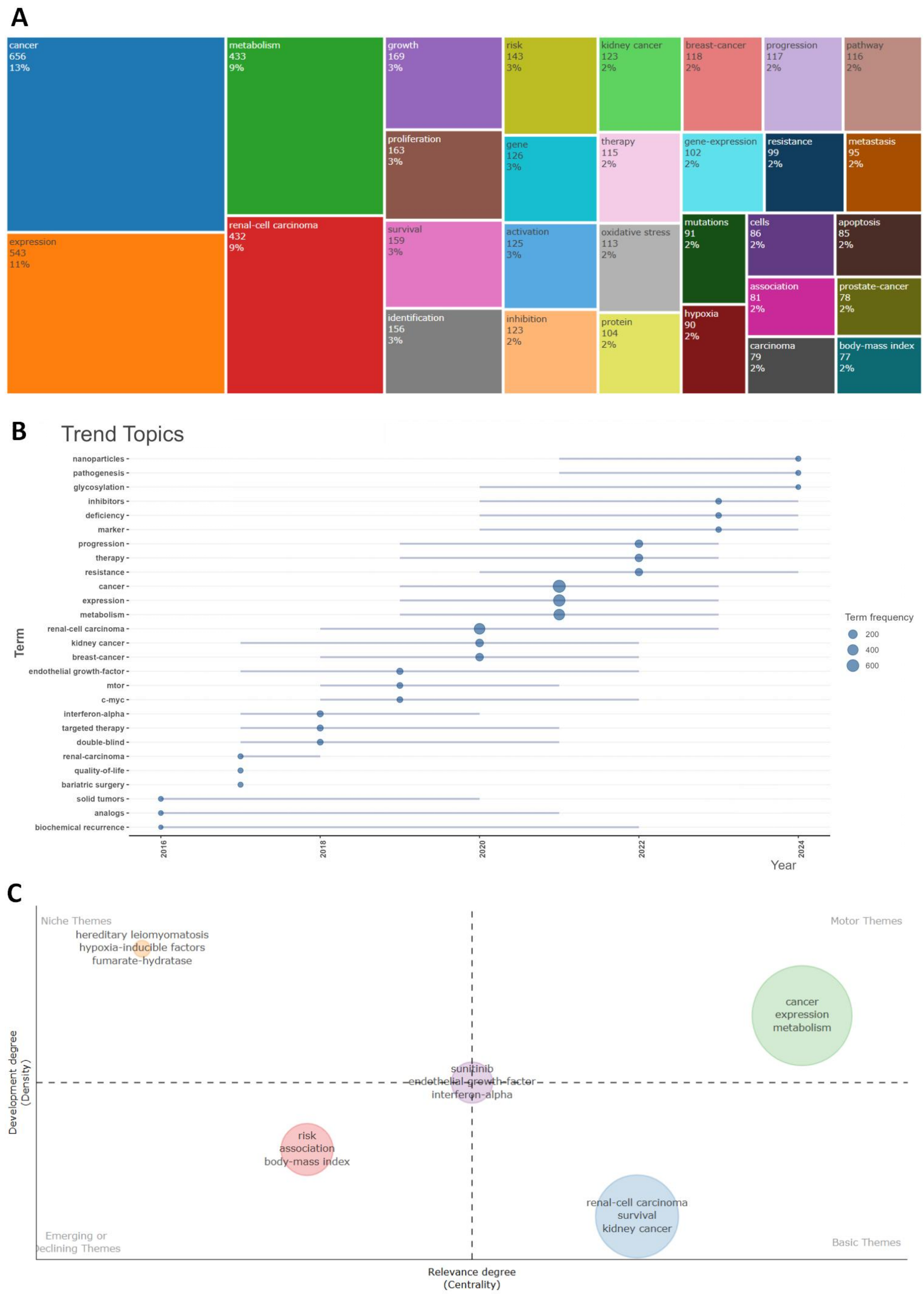

Figure S4. Keyword and hot spot analysis. (A) “Cancer”, “renal cell carcinoma”, “expression”, “metabolism” were top 4 keywords with frequency over 500. (B) The dynamic plot demonstrates the development of trend topics overtime. (C) The thematic map classified themes into four groups with two dimensions. The “Density” axis represented the development degree and the “Centrality” axis signified the relevance degree of these themes or fields.

Table S1. The top 20 most productive journals.

Rank	Sources	N. of publications	TCs	H index	IF [2023]	Quartile in category
1	CANCERS	83	1205	20	4.5	Q1
2	INTERNATIONAL JOURNAL OF MOLECULAR SCIENCES	79	2186	24	4.9	Q1
3	FRONTIERS IN ONCOLOGY	77	932	17	3.5	Q2
4	SCIENTIFIC REPORTS	69	1139	20	3.8	Q1
5	BMC CANCER	32	453	14	3.4	Q2
6	PLOS ONE	30	518	14	2.9	Q1
7	CELL DEATH & DISEASE	29	966	17	8.1	Q1
8	ONCOTARGET	29	951	17	5.2	Q2
9	FRONTIERS IN IMMUNOLOGY	27	575	13	5.7	Q1
10	CANCER RESEARCH	26	1090	18	12.5	Q1
11	CELLS	25	463	11	5.1	Q2
12	AGING-US	25	535	9	3.9	Q2
13	FRONTIERS IN PHARMACOLOGY	24	546	10	4.4	Q1
14	ONCOLOGY LETTERS	23	329	12	2.5	Q3
15	METABOLITES	23	405	11	3.5	Q2
16	FRONTIERS IN GENETICS	22	284	9	2.8	Q2
17	JOURNAL OF CANCER	21	311	12	3.3	Q2
18	FRONTIERS IN CELL AND DEVELOPMENTAL BIOLOGY	19	488	10	4.6	Q2
19	MEDICINE	19	94	6	1.4	Q2
20	NATURE COMMUNICATIONS	18	1637	14	14.7	Q1

TCs, total citations; IF, impact factor.

Table S2. The top 20 highly-local cited references.

Rank	Cited References	Local Citations
1	CREIGHTON CJ, 2013, NATURE	288
2	HAKIMI AA, 2016, CANCER CELL	244
3	HANAHAN D, 2011, CELL	231
4	HSIEH JJ, 2017, NAT REV DIS PRIMERS	228
5	WETTERSTEN HI, 2017, NAT REV NEPHROL	182
6	SUNG H, 2021, CA-CANCER J CLIN	181
7	LINEHAN WM, 2010, NAT REV UROL	140
8	WETTERSTEN HI, 2015, CANCER RES	140
9	HEIDEN MGV, 2009, SCIENCE	131
10	MOCH H, 2016, EUR UROL	130
11	DU WN, 2017, NAT COMMUN,	125
12	SUBRAMANIAN A, 2005, P NATL ACAD SCI USA	124
13	WARBURG O, 1956, SCIENCE	118
14	ANONYMOUS, 2020, CA CANCER J CLIN	110
15	SATO Y, 2013, NAT GENET	108
16	RINI BI, 2009, LANCET	102
17	CAPITANIO U, 2019, EUR UROL	94
18	LI B, 2014, NATURE	92
19	LINEHAN WM, 2016, NEW ENGL J MED	88
20	QIU B, 2015, CANCER DISCOV	88
